# Effect of situation similarity on younger and older adults’ episodic simulation of helping behaviours

**DOI:** 10.1038/s41598-023-36189-y

**Published:** 2023-06-06

**Authors:** A. Dawn Ryan, Ronald Smitko, Karen L. Campbell

**Affiliations:** Department of Psychology, Brock University, 1812 Sir Isaac Brock Way, St. Catharines, ON L2S 3A1 Canada

**Keywords:** Cognitive ageing, Problem solving

## Abstract

Similar cognitive processes enable us to remember the past (i.e., episodic memory) and simulate future events (i.e., episodic simulation). In the current study, we demonstrate an important role for previous experience when younger and older adults simulate future behaviours. Participants read short descriptions of a person in need of help in scenarios that were more familiar to either younger or older adults (e.g., dealing with dating apps vs writing a cheque). Participants either imagined helping the person or thought about the style of the story (control task), and then rated their willingness to help, scene vividness, emotional concern, and subjective use of theory of mind. Hierarchical mixed effect modelling revealed that both episodic simulation and one’s previous experience increased willingness to help, in that participants were more willing to help if they imagined helping and the situation was more familiar to them. Further, in simulated scenarios the relationship between previous experience and willingness to help was mediated by scene vividness and perspective-taking in younger adults, but only by perspective-taking in older adults. Taken together, these findings suggest that situation similarity and episodic simulation increase willingness to help, possibly via different mechanisms in younger and older adults.

## Introduction

As we engage with the world around us, we spend a considerable amount of time thinking about hypothetical scenarios. These would-be scenarios (i.e., episodic simulations) are crucial in our day-to-day lives as they enable us to plan for the future, make decisions, and solve problems^1,2^. According to the *constructive episodic simulation hypothesis,* we draw on previous experience (i.e., episodic memory) and flexibly recombine details from these events to simulate possible future events^1,2,4^. These vivid, sensory-rich imagined scenes largely rely on the same set of brain regions that are active when remembering the past, including the hippocampus, medial prefrontal cortex, lateral temporal cortices, and posterior cingulate cortex (often referred to as the default mode network)^3–6^. Moreover, damage to the medial temporal lobe has been shown to similarly affect both episodic memory for the past and one’s ability to imagine the future^7,8^, suggesting a link between the two processes.

Another line of evidence that supports the link between episodic memory and simulation is the finding that both abilities are similarly affected by age^9,10^. Compared to younger adults, older adults produce fewer rich, episodic details and more general, semantic details both when remembering the past and imagining future events^10,11^. Furthermore, older adults report less of a sense of re-living the past and pre-living the future when reconstructing past and imagining future events^12^, suggesting an impaired retrieval process that affects both memory and simulation^11,13^. Similar deficits have also been identified in higher-order tasks that require the use of episodic simulation^14–16^. For instance, when asked to generate solutions to a series of problems with predetermined outcomes (i.e., means-end problem solving), older adults produce fewer episodic-like details and this deficit is linked to the generation of fewer relevant solutions to the problems compared to younger adults^14^.

However, these findings are somewhat at odds with a growing body of research demonstrating that episodic simulation of helping behaviour (i.e., imagining helping others—another task that requires problem-solving) similarly increases willingness to help in younger and older adults^17–19^. For instance, both cohorts exhibit greater willingness to help after imagining themselves helping a person in need, relative to a semantic control task^17,18^. This effect is thought to stem from the fact that episodic simulation can make events seem more plausible and ultimately, shape one’s intentions and subsequent behaviour^2,20–23^. Moreover, in addition to increasing one’s prosocial intentions, imagining future helping scenarios also increases participants’ ratings of scene vividness similarly in both younger and older adults, as well as the degree to which they consider the thoughts and feelings of the person in need^17–19^. These phenomenological experiences are thought to mediate the effect of episodic simulation on willingness to help, in that imagining helping increases emotional concern for the person in need and vividness of the imagined scene, which in turn contribute to an increased intention to help^17–19^.

If older adults have impaired access to episodic memory, yet they exhibit changes in willingness to help following episodic simulation, it may be that older adults’ increased willingness to help arises via different mechanisms than younger adults. However, findings in this area are mixed and often at odds. For instance, Gaesser et al.^17^ showed that while subjective scene vividness similarly predicts willingness to help in both older and young adults, there was a trend for a stronger relationship between willingness to help and theory of mind in younger adults. Moreover, when they controlled for participants’ emotional concern for the person in need, the effect of episodic simulation on older adults’ willingness to help was eliminated, suggesting that their increased willingness to help is simply a result of increased emotional concern^12^. Another study using a similar paradigm found that episodic simulation increased empathic concern (i.e., the extent to which the scenario made them feel compassion, warmth, sympathy, and tenderness) to a greater degree in younger (compared to older) adults, possibly because older adults exhibited higher baseline levels of empathic concern^18^. Nevertheless, older adults exhibited an increase in willingness to help that was comparable to younger adults^18^, highlighting the need for further clarification as to whether the mechanisms of episodic simulation differ between young and older adults.

### The role of previous experience on future simulations

Given the importance of episodic memory and scene vividness on future simulations, it is important to consider how one’s previous experience with the situation may affect one’s ability to imagine future helping scenarios. Indeed, events simulated in familiar, compared to unfamiliar, locations are reported as being clearer^24^. Moreover, young adults have been shown to produce more semantic-like details when imagining unfamiliar events^25^, and this work has recently been extended to older adults^19^. In a recent series of experiments, Ryan et al.^19^ examined whether episodic simulation could increase younger and older adults’ willingness to help in unfamiliar scenarios posed by the COVID-19 pandemic. Participants read a series of short scenarios, half of which depicted people in need in everyday situations, and half depicted COVID-related problems. Notably, data for this project was collected in the early days the pandemic, when COVID was largely unfamiliar. Results showed that imagining helping a person in need increased younger and older adults’ willingness to help in both everyday and COVID-related scenarios, though participants were less willing to help overall in the less familiar (and potentially riskier) COVID scenarios. Moreover, both groups produced more semantic-like details when imagining COVID-related scenarios, suggesting that people may need to draw on more semantic knowledge than personal experience to simulate unfamiliar scenarios.

### The current study

While Ryan et al.^19^ showed that both older and younger adults can simulate helping in novel COVID-related scenarios, the more familiar “everyday” scenarios used in that study were ones previously developed *by* young-adult researchers *for* young-adult participants^23^. Beyond pure memory abilities, the type of stimuli to be remembered or simulated likely plays a role in whether age differences in memory or future simulation are observed^26,27^. It is possible that age differences in episodic simulation may be even less pronounced if using problem scenarios developed with and tailored for each group. Furthermore, COVID-related scenarios also differed from everyday scenarios in terms of the threat posed by the contagion and concerns about social distancing. Thus, the goal of the current study was to test whether manipulating each age group’s familiarity with more typical problem scenarios would affect their ability to simulate the event and, as a result, their willingness to help. Much of the research examining episodic simulation of unfamiliar scenarios has manipulated scene familiarity by asking participants to imagine scenarios occurring at either familiar or unfamiliar settings^28^. Thus, in many cases, research examining episodic simulation of familiar and unfamiliar events requires participants to imagine events occurring under largely improbable conditions (e.g., climbing Mount Everest)^24,25,29^, which may be especially implausible to older adults. Furthermore, research examining older adults’ ability to imagine unfamiliar scenarios is limited, and extant research suggests that both younger and older adults experience more subjective detail in events imagined in more familiar spatial contextual cues (i.e., familiar landmarks^28^).

To determine which scenarios were most/least familiar to older and younger adults, we first consulted with each age group to develop a list of scenarios potentially familiar to each cohort. We then ran a pilot study in which older and younger adults rated their familiarity with each scenario. Twelve scenarios that showed a large difference in familiarity between younger and older adults (6 younger-familiar, 6 older-familiar) were selected for the main experiment. In this experiment, participants read a series of problem scenarios and were instructed to either imagine themselves helping the person in need, or to judge the journalistic source and style of the story. For each story, participants rated their willingness to help the person in need and their phenomenological experiences (i.e., scene vividness, perspective-taking, and emotional concern).

We expected both older and younger adults to show increased willingness to help following episodic simulation relative to the journalistic style condition. Further, given the role of episodic memory in future simulations, we expected this effect to be moderated by similarity ratings (i.e., a greater increase in willingness to help for scenarios that were rated as more similar to one’s previous experiences). We also expected higher similarity ratings to be related to higher ratings of scene vividness, as these are scenarios for which participants likely have memories^30^. Moreover, previous work using the current paradigm has demonstrated that scene vividness may be a mechanism through which episodic simulation influences willingness to help^17–19,23,31^. Thus, we expected an indirect relationship between story similarity (to one’s personal experience) and willingness to help via scene vividness, such that increased situation similarity increases the vividness of a scene in one’s mind, which in turn increases one’s willingness to help. Finally, given well-established age differences in episodic memory, we expected age to moderate the path between similarity and scene vividness, such that older adults should exhibit a reduced relationship between previous event similarity and scene vividness.

## Methods

### Scenario pilot

To ensure the stimuli used in our experiment contained topics that were both familiar and unfamiliar to younger and older adult, we piloted 40 potential scenario topics in an online study run using the Qualtrics’ Research Panel. Participants included 67 younger (*M* = 28.36, *SD* = 4.99, range = 18–35) and 68 older (*M* = 70.25, *SD* = 4.33, range = 60–80) Canadian residents. Among younger adults, 45.6% self-identified as White, Caucasian, or European, 23.5% as Asian, 5.9% as Canadian (including French Canadian), 5.9% as Middle Eastern, 4.4% as Black, African American/Canadian or African, 4.4% as Indian (including South India), 1.5% as mixed ethnicity, 1.5% as Hispanic or Latin American, 1.5% as Native American, and 5.9% as unknown or refused to answer. Among older adults, 67.6% self-identified as White, Caucasian, or European, 22.1% as Canadian (including French Canadian), 5.9% as Asian, 1.5% as Indian (including South India), 1.5% as Native American, and 1.5% as unknown or refused to answer.

The piloted scenarios included those used in previous research^31^ as well as scenarios created in consultation with younger and older adults. Participants rated each scenario topic on how familiar it was (1 unfamiliar–7 very familiar), how similar it was to events they had experienced in the past (1 not at all–7 very similar), and then completed a brief demographic questionnaire. We selected the 6 stories rated most familiar and similar to events experienced by younger and older adults (12 stories total, provided in the Supplementary Information). The selected stories were then divided into lists of young-familiar and old-familiar stimuli that differed significantly between age groups in terms of similarity, *t*_*older-familiar*_ (134) = 3.80, *p* < 0.001, *t*_*younger-familiar*_ (134) = 6.81, *p* < 0.001, and familiarity, *t*_*older-familiar*_ (134) = 3.20, *p* = 0.002, *t*_*younger-familiar*_ (134) = 5.74, *p* < 0.001, (see Table 1 for scenario list means). Raw participant ratings of all 40 scenarios as well as their demographics are available at (https://osf.io/wzgvf/?view_only=136eccd317104423be4d77a18cfbab61).

**Table 1:** Participant ratings of scenario lists by age.

Scenario list	Ratings			
	Similarity		Familiarity	
Pilot data	YA	OA	YA	OA
Younger-Familiar	3.79 (1.55)	2.19 (1.16)	3.95 (1.64)	2.45 (1.40)
Older—familiar	3.84 (1.43)	4.77 (1.44)	4.07 (1.52)	4.98 (1.47)
Main experiment				
Younger-Familiar	3.73 (1.52)	2.51 (1.36)	4.26 (1.48)	3.19 (1.52)
Older-familiar	3.54 (1.55)	3.68 (1.46)	3.87 (1.52)	4.32 (1.41)

*Note* Standard deviation in parentheses.

### Main experiment participants

We aimed to test the same number of participants as our previous study using a similar paradigm^19^: 100 younger (18–35 years) and 100 older (60–80 years) adults. Participants were recruited through the Qualtrics’ Research Panel and included both Canadian and American residents. All participants reported being fluent in English with no history of stroke, neurological conditions (e.g., epilepsy), cognitive impairment (e.g., dementia, Alzheimer’s) or psychiatric issues (e.g., schizophrenia or bipolar disorder). In total, 223 participants completed the study, and 7 study responses were removed due to having duplicate IP addresses. A further 21 participants were removed for having too few correctly performed trials (see below). Finally, 12 older and 9 younger adults were removed due to poor performance on the adapted version of the Mini-Mental State Exam (MMSE)^32^.

Participants’ open-ended responses were used to assess whether each trial was completed correctly. Incorrect trials were defined as those in which participants explicitly mentioned performing the opposite task (e.g., judging the journalistic style of a story when they were supposed to be imagining helping). Incorrect trials were excluded from the analysis, and participants with > 50% of trials incorrect were excluded from the study all together^19^.

After data cleaning, 83 younger (*M* = 22.88, *SD* = 3.50, 63.9% women, 1.2% non-binary) and 91 older adults (*M* = 70.88, *SD* = 4.69, 53.8% women) with usable data remained for analysis. Among younger adults, 47% self-identified as White, Caucasian, or European, 13.3% as Asian, 18.1% as Black, African American/Canadian or African, 2.4% as Canadian (including French Canadian), 2.4% as mixed ethnicity, 2.4% as Middle Eastern, 6% as Hispanic or Latin American, 1.2% as Caribbean, 1.2% as Native American, and 6% as unknown or refused to answer. Among older adults, 75.8% self-identified as White, Caucasian, or European, 15.4% as Canadian or American, 3.3% as Asian, 2.2% as Black, African American/Canadian or African, 1.1% as Middle Eastern, 1.1% as Hispanic or Latin American, and 1.1% as Indian. To determine the observed power in our final, cleaned sample (n = 174), we conducted a post-hoc power analysis using G*Power, where alpha = 0.05 and the correlation between repeated measures = 0.49^33^. Results indicate that a power of 0.887 was achieved to detect the age × condition interaction. However, it is important to note that post-hoc power calculations that derive effect sizes from the collected data can be misleading^34^.

Participants from the pilot and experimental studies gave informed consent to participate and were free to exit the studies at any time. The present research was approved by the Research Ethics Board of Brock University (21-034), and all research was conducted in accordance with the approved guidelines/regulations.

### Experimental procedure

The present study adapted the paradigm used in previous research on episodic simulation of helping behaviour^17–19,31^. Participants read one-line stories depicting examples of people in need of help. Half of the stories described events that were similar to those previously experienced by younger adults (e.g., “After a day out with friends, this person sees themself tagged in an unflattering photo online.”), while the other half were similar to those previously experienced by older adults (e.g., “This person has found out they have not saved enough for their retirement.”). In separate blocks, participants were asked to either: (1) focus on the story by considering its journalistic style and online media source (No Helping condition) or (2) imagine a vivid scenario of helping the person in need (Imagine Helping condition). Block order and stories were counterbalanced across participants.

At the beginning of each block participants completed a practice trial and were asked whether they understood the instructions. Anyone who indicated they did not understand the instructions were provided with further instructions/examples, and those who reported understanding the task were immediately forwarded to the experiment. Anyone who did not understand the instructions after two checks was screened out of the study.

On each trial, the story was displayed in the centre of the screen for 10 s followed by the condition prompt. During the condition prompt, participants provided a written description of their imagined scenario or how they judged the style of the story. Each prompt lasted a minimum of 60 s, but participants were able to continue writing if they wanted more time. Participants then completed self-paced ratings of how willing they would be to help the person in need (1 = not at all–7 = very willing), scene coherence (1 = vague–7 = coherent and clear) and detail (1 = simple–7 = detailed) in their mind, whether the story made them feel troubled, distressed, sympathetic, compassionate, worried, and moved (1 = not at all–7 extremely for each emotion separately), and how much they considered the thoughts and feelings of the person in need (i.e., perspective-taking; 1 = not at all–7 = a great deal). Participants also rated each scenario on how similar it was to situations they have previously experienced (1 = not at all–7 = very similar). Participants completed 12 trials in total, with 3 younger-familiar and 3 older-familiar scenarios stories in each condition (Imagine Helping vs No Helping). Because the vividness of imagined future events can be influenced by individual differences in visual imagery capacity^21^, participants completed the Vividness of Visual Imagery Questionnaire (VVIQ)^35^. There was no significant difference between younger (*M* = 76.63, *SE* = 2.66) and older (*M* = 69.84, *SE* = 2.61) adults’ scores on the VVIQ, *t* (172) = 1.82, *p* = 0.071, suggesting that self-reported mental imagery ability was similar between groups. Finally, participants completed a version of the Mini-Mental State Examination (MMSE)^36^ that has been adapted for remote administration^32^, and a demographics questionnaire.

### Analytic plan

The present study builds on previous work by using hierarchical mixed effect modeling to conduct trial-wise, rather than participant-wise analyses (i.e., an ANOVA for primary^17,18^ or exploratory analyses^18,19^). This also allowed us to use participants’ actual ratings of situation similarity on a situation-by-situation basis, rather than dichotomizing the variable^19,28^, which allows us to take individual experience into account.

We used the lmer package in R to construct hierarchical mixed effects models. To account for individual differences, we included participant id as a random effect in the model. Further, because the stimuli in the current study may differ in other ways apart from similarity to one’s previous experiences (e.g., social vs non-social tasks), we also included story as a random effect when constructing the initial model. Random effects and fixed factors were added to the model one at a time in the following order: (1) Condition, (2) Similarity Ratings, (3) Condition × Similarity Ratings, (4) Age, (5) Condition × Age, (6) Similarity Ratings × Age, and (7) Age × Condition × Similarity Ratings. These 7 models were compared, and the best fit model was constructed by including only predictors that improved model fit^37^. Follow-up t-tests and mixed effects models were conducted to explore the nature of interactions when appropriate. To examine potential mechanisms of episodic simulation in younger and older adults, moderated mediation analyses were conducted using the “MLMED" macro^38^. Specifically, on episodic simulation trials only, we tested whether the relationship between similarity and willingness to help was moderated by different phenomenological experiences (i.e., scene vividness, emotional concern, and perspective-taking) in each age group. Participants’ willingness to help ratings were entered as the dependent variable, situation similarity ratings the independent variable, phenomenological experiences as potential mediators, and age group as a potential moderator. Follow-up mediation analyses within each age group were then performed for any effects that were significantly moderated by age.

## Results

### Situation similarity ratings

As a manipulation check, we first conducted a 2 (Story Type: Younger Familiar vs. Older Familiar) X 2 (Age: Younger vs. Older Adults) repeated measures ANOVA on participants’ ratings of situation similarity. There was a main effect of story, *F* (1, 172) = 34.56, *p* < 0.001, *ηp*^2^ = 0.167, due to participants rating older-familiar (*M* = 3.61, *SE* = 0.11) stories as being more similar to situations they had previously experienced than the younger-familiar stories (*M* = 3.12, *SE* = 0.11) overall. There was also a main effect of age, *F* (1, 172) = 6.78, *p* = 0.010, *ηp*^2^ = 0.038, due to younger adults (*M* = 3.63, *SE* = 0.15) rating all scenarios as more similar to those they had previously experienced than older adults (*M* = 3.09, *SE* = 0.14). Finally, we found a significant story type by age interaction, *F* (1, 172) = 66.45, *p* < 0.001, *ηp*^2^ = 0.279. Pairwise t-tests confirmed that older adults rated older-familiar (*M* = 3.68, *SE* = 0.15) stories as being more similar to those they had previously experienced than younger-familiar stories (*M* = 2.51, *SE* = 0.14), *t* (90) = 9.58, *p* < 0.001, and younger adults rated younger-familiar stories (*M* = 3.73, *SE* = 0.17) as being more similar to those they had previously experienced than older-familiar stories (*M* = 3.54, *SE* = 0.17), *t* (82) = 1.69, *p* = 0.047. Thus, both groups rated their own-group stories as being more similar to their previous experiences than other-group stories. It should be noted that while we performed a manipulation check by treating story similarity as a categorical variable with two levels (i.e., old-familiar, young-familiar), the following analyses use participants’ individual ratings of situation similarity as these are more specific to each individual’s previous experiences and can take cross-trial variability into account.

### Effects of condition and story similarity

#### Willingness to help

**Figure 1:**
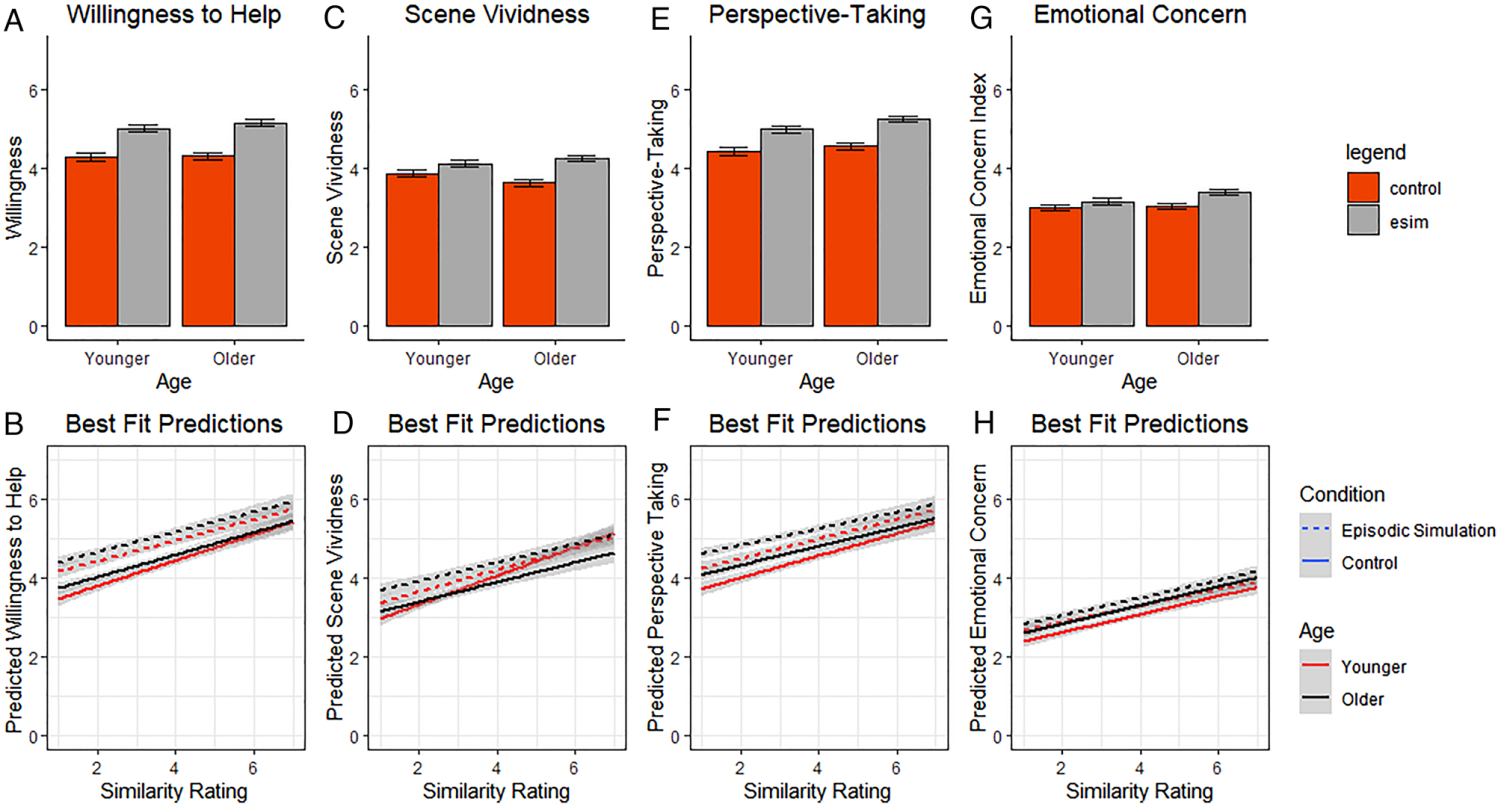
Observed means of participants’ ratings and best fit model predictions across conditions. *Note* Error bars and shaded portions represent standard error of the mean.

The model predicting willingness to help included participant id (ICC = 0.46) and story number, *χ2*(1) = 69.59, *p* < 0.001; ICC = 0.50, as random effects. Condition, *χ2*(1) = 143.13, *p* < 0.001, and similarity, *χ2*(1) = 124.02, *p* < 0.001, were found to improve model fit and were retained for the best fit model. All other predictors did not improve model fit, *p’s* > 0.118. The best fit model for willingness to help revealed an effect of condition, with participants reporting a greater willingness to help following episodic simulation (*M* = 5.06, *SE* = 0.13) compared to the control condition (*M* = 4.34, *SE* = 0.14; see Fig. 1A for observed means and Table 2 for best fit estimates). The best fit model also revealed a positive effect of similarity, such that higher levels of situation similarity were related to a greater willingness to help overall (see Fig. 1B).

**Table 2:** Best fit models.

Predictors	Willingness			Scene vividness			Emotional concern			Perspective-taking		
	Estimates	CI	p	Estimates	CI	p	Estimates	CI	p	Estimates	CI	p
(Intercept)	3.71	3.42–3.99	** < 0.001**	3.20	2.83–3.56	** < 0.001**	2.63	2.37–2.89	** < 0.001**	4.04	3.78–4.29	** < 0.001**
Condition	0.73	0.60–0.85	** < 0.001**	0.20	0.05–0.35	**0.011**	0.23	0.13–0.34	** < 0.001**	0.57	0.45–0.69	** < 0.001**
Similarity	0.19	0.16–0.23	** < 0.001**	0.21	0.17–0.25	** < 0.001**	0.12	0.09–0.15	** < 0.001**	0.15	0.12–0.18	** < 0.001**
Control trials: age contrast				0.23	− 0.24 to 0.70	0.341						
Simulated Trials: Age Contrast				0.53	0.06–1.00	**0.029**						
Similarity: Age Contrast				− 0.11	− 0.16 to − 0.05	** < 0.001**						
Random effects												
σ^2^	1.71			1.16			1.30			1.60		
τ_00_	1.53_Participant ID_			1.87_Participant ID_			1.07_Participant ID_			1.56_Participant ID_		
	0.09_Story #_			0.03_Story #_			0.09_Story #_			0.04_Story #_		
ICC	0.49			0.62			0.47			0.50		
N	172_Participant ID_			172_Participant ID_			172_Participant ID_			172_Participant ID_		
	12_Story #_			12_Story #_			12_Story #_			12_Story #_		
Observations	1854			1854			1854			1854		
Marginal R^2^/Conditional R^2^	0.092/0.533			0.051/0.641			0.037/0.491			0.062/0.532		

*Note* Contrasts reflect the comparison to the control condition and younger adults.

Significant values are in bold.

#### Scene vividness

Scene vividness was calculated by averaging participants’ ratings of coherence and detail on each trial^23^. In terms of predicting scene vividness ratings, both participant id (ICC = 0.62) and story number, *χ2*(1) = 17.21, *p* < 0.001; ICC = 0.63 were retained as random factors. Condition, *χ2*(1) = 50.30, *p* < 0.001, similarity, *χ2*(1) = 104.06, *p* < 0.001, the condition by age interaction, *χ2*(1) = 6.90, *p* = 0.008, and the similarity by age interaction, *χ2*(1) = 14.725, *p* < 0.001 were found to improve model fit and were retained for the best fit model. All other predictors did not improve model fit, *p’s* > 0.307.

The best fit model for scene vividness revealed an effect of condition, with participants reporting higher scene vividness ratings following episodic simulation (*M* = 4.17, *SE* = 0.12) compared to the control condition (*M* = 3.82, *SE* = 0.12; see Fig. 1C for observed means and Table 2 for best fit estimates). There was also an effect of similarity, such that higher similarity ratings were related to increased scene vividness ratings (see Fig. 1D). The similarity by age interaction was due to the relationship between similarity and scene vividness being attenuated in older adults (see below). The condition by age interaction was due to an age effect in the imagined, but not control condition. A follow-up analysis revealed that although the effect of condition increased scene vividness ratings for both younger, *t*(1680) = 2.55 *p* = 0.011, and older adults, *t*(1675) = 7.25 *p* < 0.001, the change was more pronounced in older adults.

To further explore the interaction between similarity and age on scene vividness, we constructed best fit models separately within each age group. In younger adults, both participant id (ICC = 0.53) and story number, *χ2*(1) = 6.61, *p* = 0.01; ICC = 0.55 were retained as random factors. Condition, *χ2*(1) = 6.76, *p* = 0.009 and similarity, *χ2*(1) = 80.48, *p* < 0.001, were found to improve model fit and were retained for the best fit model. The condition by similarity interaction did not improve model fit, *p* = 0.491.

The best fit model for scene vividness ratings in younger adults confirmed there was an effect of condition, *B* = 0.19, *SE* = 0.08, *t*(740.92) = 2.30, 95% *CI* [0.03, 0.36], with younger participants reporting greater scene vividness following episodic simulation (*M* = 4.13, *SE* = 0.16) compared to the control condition (*M* = 3.94, *SE* = 0.17). There was also a positive effect of similarity, *B* = 0.22, *SE* = 0.02, *t*(804.22) = 9.22, 95% *CI* [0.17, 0.27], such that higher levels of situation similarity ratings were related to greater scene vividness. Random effects for the best fit model were σ^2^ = 1.40, ICC = 0.52, τ_00 id_ = 1.47, τ_00 Story Number_ = 0.06. Marginal and Conditional R^2^ for the model were 0.076 and 0.559, respectively.

In older adults, both participant id (ICC = 0.69) and story number, *χ2*(1) = 6.61, *p* = 0.01; ICC = 0.70 were retained as random factors. Condition, *χ2*(1) = 68.10, *p* < 0.001 and similarity, *χ2*(1) = 27.85, *p* < 0.001, were found to improve model fit and were retained for the best fit model. The condition by similarity interaction did not improve model fit, *p* = 0.450.

The best fit model for scene vividness ratings in older adults confirmed there was an effect of condition, *B* = 0.49, *SE* = 0.06, *t*(930) = 7.94, 95% *CI* [0.37, 0.61], with older participants reporting greater scene vividness following episodic simulation (*M* = 4.23, *SE* = 0.17) compared to the control condition (*M* = 3.74, *SE* = 0.17). There was also a positive effect of similarity *B* = 0.09, *SE* = 0.02, *t*(682) = 5.33, 95% *CI* [0.06, 0.12], such that higher levels of situation similarity ratings were related to greater scene vividness. Random effects for the best fit model were σ^2^ = 0.95, ICC = 0.70, τ_00 id_ = 2.23, τ_00 Story Number_ = 0.01. Marginal and Conditional R^2^ for the model were 0.033 and 0.711, respectively. Thus, the interaction between similarity and age on scene vividness in the combined model is due to the effect of similarity being significantly attenuated in the older group.

#### Perspective-taking

In terms of predicting perspective-taking, both participant id (ICC = 0.49) and story number, *χ2*(1) = 32.42, *p* < 0.001; ICC = 0.51 were retained as random factors. Condition, *χ2*(1) = 98.48, *p* < 0.001, similarity, *χ2*(1) = 84.59, *p* < 0.001, and the condition by similarity interaction, *χ2*(1) = 4.10, *p* = 0.042 were found to improve model fit and were retained for the best fit model. All other predictors did not improve model fit, *p’s* > 0.133.

The best fit model for perspective-taking revealed an effect of condition, with participants reporting greater consideration of the thoughts and feelings of the individual (i.e., perspective-taking) following episodic simulation (*M* = 5.11, *SE* = 0.12) compared to the control condition (*M* = 4.54, *SE* = 0.12; see Fig. 1E for observed means). The best fit model also revealed a positive effect of similarity, such that higher levels of situation similarity ratings related to greater perspective-taking (see Fig. 1F). However, the interaction between similarity and condition revealed that the relationship between similarity and perspective-taking was significantly attenuated in the imagined condition, *B* = − 0.06, *SE* = 0.03, *t*(1700.60) = 2.03, 95% *CI* [− 0.11, − 0.00].

#### Emotional concern

In keeping with previous research, emotional concern was calculated by averaging participants’ ratings of emotions felt on each trial^19,23^ (see SI for a similar pattern of results using empathic concern). In terms of predicting emotional concern ratings, both participant id (ICC = 0.45) and story number, *χ2*(1) = 94.38, *p* < 0.001; ICC = 0.49 were retained as random factors. Condition, *χ2*(1) = 23.53, *p* < 0.001 and similarity, *χ2*(1) = 68.33, *p* < 0.001, were found to improve model fit and were retained for the best fit model. All other predictors did not improve model fit, *p’s* > 0.093.

The best fit model for emotional concern revealed an effect of condition, with participants reporting greater emotional concern for the person in need following episodic simulation (*M* = 3.27, *SE* = 0.13) compared to the control condition (*M* = 3.03, *SE* = 0.12; see Fig. 1G for observed means). The best fit model also revealed a positive effect of similarity, such that higher levels of situation similarity ratings were related to greater emotional concern (see Fig. 1H).

### Modeling the effect of similarity on willingness to help through phenomenological experience

**Figure 2:**
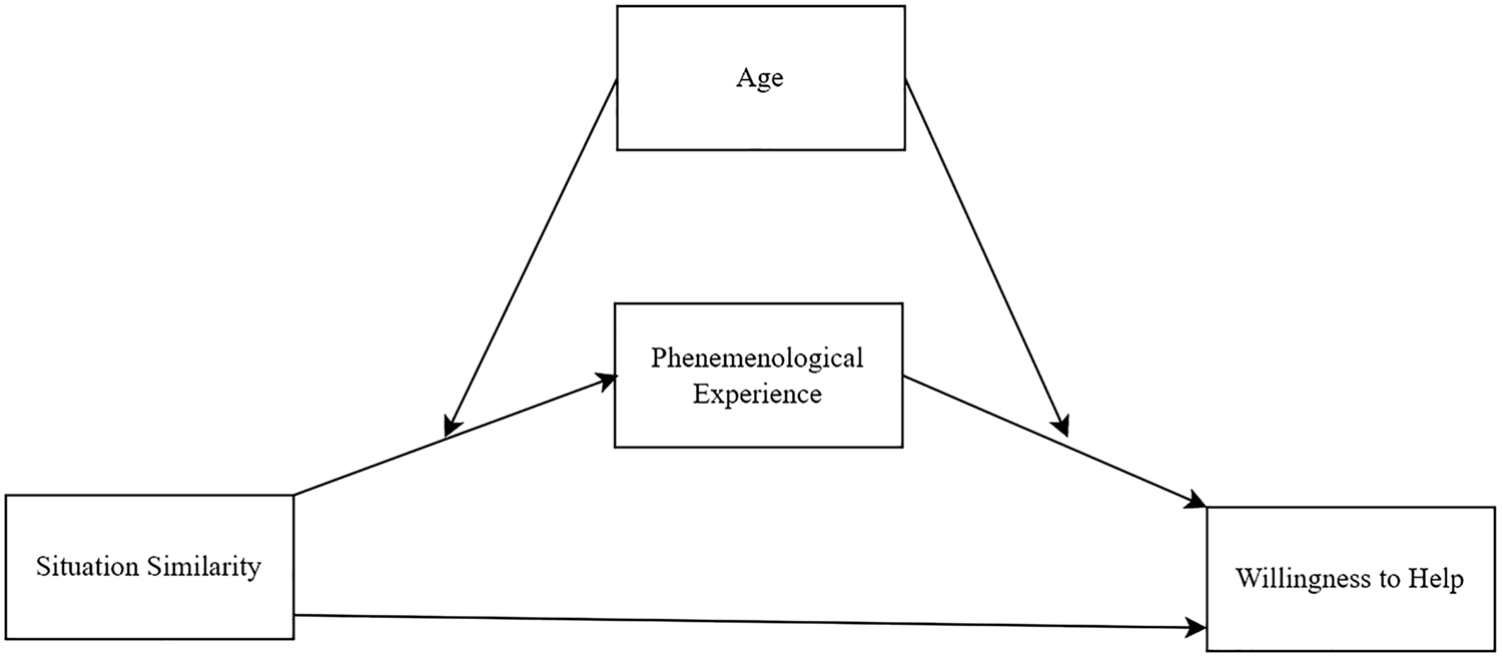
Conceptual diagram of the indirect effect of situation similarity on willingness to help, moderated by age. *Note* Phenomenological experience refers to scene vividness, perspective-taking, and emotional concern. Each phenomenological experience was initially explored as a potential mediator in separate models. Significant mediators were then tested in parallel.

To further examine how one’s previous experiences influence willingness to help in younger and older adults following episodic simulation, we conducted a series of moderated mediation analyses on simulated trials in which willingness to help was the dependent variable, situation similarity the independent variable, phenomenological experiences the potential mediators (one at a time), and age group a potential moderator (see Fig. 2 for a conceptual diagram). Because episodic memory can influence cognitive and affective empathy^39^ we also tested whether the relationship between episodic simulation and willingness to help was mediated by perspective taking (i.e., cognitive empathy) and/or emotional concern (i.e., affective empathy) while controlling for scene vividness (see Supplementary Information).

For scene vividness, we found an indirect effect of situation similarity on willingness to help via scene vividness, effect = 0.06, SE = 0.01, 95% *CI* (0.04, 0.09), suggesting that situation similarity influences scene vividness, which in turn influences participants’ willingness to help. As expected, we also found a significant interaction between age and similarity on scene vividness, effect = − 0.10, SE = 0.04, 95% *CI* (− 0.18, − 0.03), suggesting that the path between similarity and scene vividness differs between younger and older adults. This was confirmed by the index of moderated mediation, estimate = − 0.03, 95% *CI* (− 0.07, − 0.01). There was no interaction between age and scene vividness on willingness to help, effect = − 0.04, SE = 0.08, 95% *CI* (− 0.20, 0.11), suggesting that the path between scene vividness and willingness to help did not differ across age groups. This was confirmed by the index of moderated mediation, estimate = − 0.01, 95% *CI* (− 0.04, 0.03). Follow-up mediation analyses within each age group revealed that there was a stronger indirect effect of scene vividness in younger adults, effect = 0.07, SE = 0.02, 95% *CI* (0.04, 0.10) than in older adults, effect = 0.02, SE = 0.01, 95% *CI* (0.01, 0.04).

For emotional concern, we found an indirect effect of situation similarity on willingness to help via emotional concern, effect = 0.04, SE = 0.01, 95% *CI* (0.01, 0.07), suggesting that greater familiarity with a situation increases emotional concern, which in turn influences willingness to help. The indices of moderated mediation were not significant for the path between similarity and emotional concern, estimate = − 0.02, 95% *CI* (− 0.05, 0.02), nor emotional concern and willingness to help, estimate = − 0.002, 95% *CI* (− 0.02, 0.01), suggesting that the indirect effect was of similar magnitude in both younger and older adults (notably, these results do not change when using empathic concern as discussed in the SI).

For perspective-taking, we found an indirect effect of situation similarity on willingness to help via perspective-taking, effect = 0.05, SE = 0.02, 95% *CI* (0.01, 0.09), suggesting that greater familiarity with a situation relates to greater consideration of the thoughts and feelings of the person in need, which in turn influences willingness to help. The index of moderated mediation for the path from similarity to perspective taking was not significant, estimate = 0.02, 95% *CI* (− 0.04, 0.07). However, there was a significant interaction between age and perspective taking on willingness to help, effect = 0.13, SE = 0.06, 95% *CI* (0.02, 0.24), suggesting that the path between perspective taking and willingness to help differs between younger and older adults. This was confirmed by the index of moderated mediation, estimate = 0.01, 95% *CI* (0.0005, 0.02). Follow-up mediation analyses within each age group revealed that there was a stronger indirect effect of perspective taking in older adults, effect = 0.08, SE = 0.02, 95% *CI* (0.04, 0.11) than in younger adults, effect = 0.05, SE = 0.02, 95% *CI* (0.01, 0.09).

Since all three phenomenological experiences showed similar mediating effects, we finally ran parallel mediation models in younger and older adults separately with scene vividness, emotional concern, and perspective-taking entered into each model together. In younger adults, the paths through scene vividness, effect = 0.03, SE = 0.01, 95% *CI* (0.01, 0.05), and perspective-taking, effect = 0.04, SE = 0.02, 95% *CI* (0.01, 0.08), were both significant. The indirect path through emotional concern was not significant, effect = 0.01, SE = 0.01, 95% *CI* (− 0.01, 0.02). In older adults, the path through perspective-taking, effect = 0.07, SE = 0.02, 95% *CI* (0.04, 0.11) was significant. However, the indirect effects of scene vividness, effect = 0.01, SE = 0.004, 95% *CI* (− 0.002, 0.01) and emotional concern, effect = 0.002, SE = 0.003, 95% *CI* (− 0.002, 0.11) were not significant. Taken together, these findings suggest that following episodic simulation, one’s previous experience influence willingness to help via different mechanisms in younger and older adults.

## Discussion

The present study explored how situation similarity influences episodic simulation of helping behaviour in younger and older adults. We demonstrate that story similarity ratings (i.e., how similar a situation is to those previously experienced) influences one’s willingness to help overall, with people being more willing to help in situations that are similar to those they have personally experienced. Further, episodic simulation brought about cognitive change by increasing willingness to help, scene vividness, emotional and empathic (see SI) concern, and perspective-taking relative to a baseline condition. Notably, the lack of age difference in these effects suggests that older adults can engage in episodic simulation to a similar degree as younger adults. However, exploration of previously identified mechanisms through which episodic simulation affects prosocial intentions^18,31^ revealed that these mechanisms seem to differ between younger and older adults. In the present study, story similarity was found to act via different mechanisms in younger and older adults, with younger adults showing indirect effects through scene vividness and perspective-taking, while older adults showed an indirect effect through perspective-taking only. Strikingly, the indirect paths seemed to be influenced by age at different stages. Specifically, advanced age had a negative effect on the relationship between situation similarity and scene imagery, but a positive effect on the relationship between perspective taking and willingness to help. Notably, in parallel mediation models, both younger and older adults did not show an indirect effect of emotional concern. Taken together, these findings suggest that episodic simulation is relatively preserved with age, and that personal experience with similar situations influences simulated outcomes, such as willingness to help, via different mechanisms in younger and older adults.

Previous work exploring how one’s lived experiences affect episodic simulation has typically contrasted common, everyday scenarios with largely hypothetical events (e.g., climbing Mount Everest)^24,25,29^ or manipulated familiarity of the imagined location itself^28,31^. Critically, the work here uses a series of stimuli that were created in consultation with older adults, and piloted on an initial sample, to help ensure suitable variability in how similar stories were to older adults’ previous experiences (i.e., ensuring half the stories were similar and half were dissimilar to the typical experiences of older adults). Further, the analytical approach used in the current study also takes into account participants’ individual ratings of situation similarity (treating similarity as a continuous, rather than as a discrete, measure). By using real-world scenarios that are familiar to either older or younger adults and taking a trial-by-trial approach to the data, the current study contributes to the literature by showing how one’s previous experience influences episodic simulation of helping behaviour in older adults.

Although episodic simulation may similarly increase willingness to help in both older and younger adults, recent work suggests that baseline levels of prosociality, and concern for the ‘greater good’, may be higher in older adults^18,40^. Indeed, compared to younger adults, older adults show less of a self-serving bias, in that they expend a similar amount of effort to help others as they do to benefit themselves^41^. Moreover, although some research indicates that older adults exhibit reduced cognitive empathy (i.e., perspective taking of others’ emotional state^42,43^, affective empathy (i.e., feeling, compassion) is relatively stable across the lifespan^44^. Such findings are in line with the *socioemotional selectivity theory*, which suggests that older adults are motivated to partake in activities that maximize emotional fulfillment and focus on the common good^40,45–47^. As such, despite the evidence for age-related decline in episodic memory and simulation, chronic goal shifts toward emotional well-being and the greater good may motivate older adults to try harder when imagining themselves helping those in need, or consider themselves as being more willing to help regardless of their simulation abilities.

### Limitations and future directions

Future research should aim to address some of the limitations of the present study. First, the current study was conducted online. While many in-lab cognitive experiments have been replicated online (including the current paradigm^19,31^), older adults who participate in online studies may be higher functioning or more computer literate than those typically tested in the lab^48^. Relatedly, the age range of older adults in the present study (60–80 years old) may include individuals who are not yet experiencing age-related cognitive decline. Thus, the current sample may consist of older adults who are not experiencing the same level of cognitive decline as those typically tested in-lab. Second, the current sample of participants consists of Canadian residents and may represent a Western, Industrialized, Educated, Rich, Democratic (WEIRD) sample^49^. Because the positivity effect may differ across cultures^50^, future research should aim to replicate the present findings in a different culture. Finally, the current findings on the mechanistic differences of episodic simulation in younger and older adults should be explored using paradigms that do not necessarily hinge on simulating helping behaviours. As discussed, older adults’ tendency to prioritize the greater good may affect their willingness to help in addition to any simulation effects, clouding potential age-related differences.

In conclusion, the present study demonstrates an important role for one’s previous experience when simulating future events. Specifically, situation similarity made significant contributions to individuals’ willingness to help, scene vividness, emotional concern, and perspective-taking. Perhaps the most striking finding here is that situation similarity affected willingness to help via different mechanisms in older and younger adults. On trials where participants imagined future helping events, older adults engaged in more perspective-taking when the helping scenarios were more similar to those they had personally experienced, and this increased their willingness to help; in contrast, younger adults were additionally better at picturing the scene and this increased their willingness to help. While episodic memory and perspective-taking share overlap^39^, it is possible to have perspective-taking without episodic memory^51^. Thus, it is conceivable that if episodic memory is inaccessible or impoverished, as it is thought to be in older adults, one’s previous experience influences future simulations via different mechanisms, especially when such events are in line with age-related shifts in chronic prosocial goals.

## Supplementary Information

Supplementary Information.

## Data Availability

The dataset from the pilot is available through the Open Science Framework and the data collected for the experimental procedure is available from the corresponding author on reasonable request.
