# Supplementary material for: Effect of situation similarity on younger and older adults’ episodic simulation of helping behaviours
Source: Sci Rep. 2023 Jun 6;13:9167. doi: 10.1038/s41598-023-36189-y (PMC10242231; doi:10.1038/s41598-023-36189-y)
Supplement: Supplementary file 1 — Supplementary Information. [file 41598_2023_36189_MOESM1_ESM.docx]

**Supplementary Information**

**Materials**

***Scenario List***

***Younger-Familiar***

- While at a party, this person suspects that their drink has been spiked.
- This person needs to select a form of birth control.
- After a day out with friends, this person sees themself tagged in an unflattering photo online.
- After making a new profile, this person has not gotten any matches on their favourite dating app.
- This person has been locked out of their email account.
- This person is being publicly ridiculed online.

***Older-Familiar***

- This person needs to use a paper map to navigate a rural town.
- This person needs to write a cheque for a friend but does not know how.
- This person has found out they have not saved enough for their retirement.
- This person needs to schedule an appointment with a new specialist.
- After the death of a family member, this person needs to plan a funeral.
- This person needs to buy home insurance and is unsure of who to contact.

**Results**

***Empathic concern****.* Because research examining younger and older adults’ episodic simulation of helping behaviour has also examined empathic concern [18], we created a composite variable by averaging *only* participants’ ratings of sympathy, compassion, and the extent to which they were moved by the stories. In terms of predicting empathic concern, both participant id (ICC = 0.47) and story number (ICC = 0.50) , *χ2*(1) = 94.38, *p* < .001, were retained as random factors. Condition, *χ2*(1) = 51.88, *p* < .001, similarity, *χ2*(1) = 102.47, *p* < .001, and age, *χ2*(1) = 7.08, *p* < .008 were found to improve model fit and were retained for the best fit model. There was also a trend towards the interaction between age and condition, *χ2*(1) = 3.86, *p* = .049, that was retained for the best fit model for exploratory purposes. All other predictors did not improve model fit, *p’s* > .167.

The best fit model for empathic concern (see Table S1) revealed an effect of condition, with participants reporting greater empathic concern for the person in need following episodic simulation (*M* = 3.94, *SE* = .13) compared to the control condition (*M* = 3.55, *SE* = .13). The best fit model also revealed a positive effect of similarity, such that higher levels of situation similarity ratings were related to greater empathic concern. Although older adults (*M* = 3.99, *SE* = .15) reported numerically higher empathic concern than younger adults (*M* = 3.49, *SE* = .16), the contrast failed to reach significance. Follow-up t-tests revealed that the interaction between age and condition was due to the effect of condition being larger in older (mean difference = 0.52, *SE* = .08, *t*(1679) = 6.36, *p*<.001) than younger adults (mean difference = 0.27, *SE* = .09, *t*(1687) = 2.92, *p* = .004). Thus, using empathic concern instead of emotional concern yielded a highly similar pattern of results.

**Table S1**

*Best fit models for emotional and empathic concern*

|  | **Emotional Concern** | | | **Empathic Concern** | | |
| --- | --- | --- | --- | --- | --- | --- |
| *Predictors* | *Estimates* | *CI* | *p* | *Estimates* | *CI* | *p* |
| (Intercept) | 2.63 | 2.37 – 2.89 | **<0.001** | 2.79 | 2.44 – 3.14 | **<0.001** |
| Condition | 0.23 | 0.13 – 0.34 | **<0.001** | 0.27 | 0.09 – 0.44 | **0.004** |
| Similarity | 0.12 | 0.09 – 0.15 | **<0.001** | 0.17 | 0.14 – 0.20 | **<0.001** |
| Effect of Age |  |  |  | 0.38 | -0.02 – 0.78 | 0.063 |
| Effect of Condition Across Age Groups |  |  |  | 0.25 | 0.01 – 0.49 | **0.040** |
| **Random Effects** | | | | | | |
| σ^2^ | 1.30 | | | 1.63 | | |
| τ_00_ | 1.07 _Participant ID_ | | | 1.44 _Participant ID_ | | |
|  | 0.09 _Story Number_ | | | 0.07 _Story Number_ | | |
| ICC | 0.47 | | | 0.48 | | |
| N | 172 _Participant ID_ | | | 172 _Participant ID_ | | |
|  | 12 _Story Number_ | | | 12 _Story Number_ | | |
| Observations | 1854 | | | 1854 | | |
| Marginal R^2^ / Conditional R^2^ | 0.037 / 0.491 | | | 0.074 / 0.519 | | |

*Note*: Contrasts reflect the comparison to the control condition and younger adults.

**Modeling the Effect of Episodic Simulation on Willingness to Help Through Emotional Concern and Perspective-Taking**

To examine whether perspective taking or emotional concern have a greater impact on willingness to help following episodic simulation of helping behaviours, we conducted moderated mediation analyses using the “MLMED" macro. For these analyses, participants’ willingness to help ratings were entered as the dependent variable, condition (control vs episodic simulation) as the independent variable, perspective taking or emotional concern as a potential mediator, and age group as a potential moderator. Because scene vividness was found to be a major contributor to willingness to help in our current and prior work [19], scene vividness was entered into the model as a covariate. Moderation was tested on each potential mediator separately at first, and follow-up mediation analyses were then performed in parallel (see Figure S1 for a conceptual diagram).


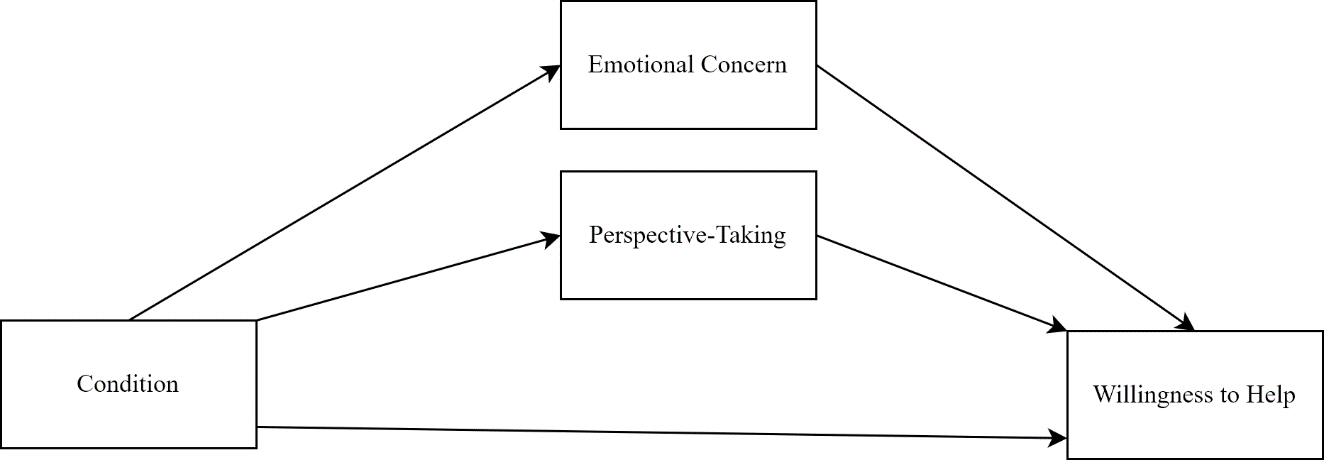
**Figure S1.***Conceptual diagram of the indirect effect of condition on willingness to help, moderated by age.*

*Note:* Each potential mediator was initially explored in separate models and subsequently tested in parallel.

For emotional concern, the indirect effect of condition on willingness to help was not significant, effect = .03, SE = .04, 95% *CI* (-.05, .10), suggesting that episodic simulation did not influence willingness to help by increasing participants’ emotional concern for the person in need. The indices of moderated mediation were not significant for the path between condition and emotional concern, estimate = .05, 95% *CI* (-.04, .15), nor emotional concern and willingness to help, estimate = -.003, 95% *CI* (-.02, .01), suggesting that the non-significant indirect effect was of similar magnitude in both younger and older adults.

For perspective-taking, we found an indirect effect of condition on willingness to help via perspective-taking, effect = .30, SE = .06, 95% *CI* (.19, .41), suggesting that simulating future helping scenarios relates to greater consideration of the thoughts and feelings of the person in need, which in turn influences willingness to help. The index of moderated mediation for the path from condition to perspective taking was not significant, estimate = -.03, 95% *CI* (-.17, .12), nor perspective-taking to willingness to help, estimate = .03, 95% *CI* (-.004, .07), suggesting that the significant indirect effect was of similar magnitude in both younger and older adults.

For exploratory purposes, we finally ran a parallel mediation model with emotional concern, and perspective-taking entered into the model as mediators and scene vividness as a covariate. The path through perspective-taking, effect = .28, SE = .04, 95% *CI* (.21, .35) was found to be significant. The indirect path through emotional concern was trending, effect = .01, SE = .01, 95% *CI* (.001, .03), but failed to reach significance (p =.056). Finally, the contrast between indirect effects was significant, effect = -.26, 95% *CI* (-,34, -.19), confirming that the indirect path via perspective-taking was stronger than the indirect path via emotional concern.
